# MoS_2_ nanosheet mediated ZnO–g-C_3_N_4_ nanocomposite as a peroxidase mimic: catalytic activity and application in the colorimetric determination of Hg(ii)†

**DOI:** 10.1039/c8ra09814j

**Published:** 2019-02-01

**Authors:** A. Anand Babu Christus, P. Panneerselvam, A. Ravikumar, M. Marieeswaran, S. Sivanesan

**Affiliations:** Department of Chemistry, SRM Institute of Science and Technology Ramapuram campus Ramapuram-600 089 Tamil Nadu India; Department of Chemistry, SRM Institute of Science and Technology Kattankulathur-603 203 Tamil Nadu India panneerselvam.pe@ktr.srmuniv.ac.in panneerchem82@gmail.com; Department of Applied Science and Technology, Anna University A. C. Tech Campus Chennai-600 025 India

## Abstract

A novel colorimetric sensing platform using the peroxidase mimicking activity of ternary MoS_2_-loaded ZnO–g-C_3_N_4_ nanocomposites (ZnO–g-C_3_N_4_/MoS_2_) has been developed for the determination of Hg(ii) ions over co-existing metal ions. The nanocomposite was prepared using an exfoliation process, and the product was further characterized using SEM, TEM, XRD and FTIR analysis. The ZnO–g-C_3_N_4_/MoS_2_ possesses excellent intrinsic catalytic activity to induce the oxidation of 3,3′,5,5′-tetramethylbenzidine (TMB) in aqueous solution in the presence of H_2_O_2_ to generate deep blue coloured cation radicals (TMB^+^) which can be viewed with the naked eye and produce absorbance at a wavelength of 652 nm. The addition of a well known bioradical scavenger, glutathione (GSH), to the solution hinders the generation of cation radicals and turns the solution colourless. The introduction of Hg(ii) to this solution brings the blue colour back into it, due to the strong affinity of the thiol in the GSH. Based on this mechanism, we have developed a simple and rapid colorimetric sensor for the highly sensitive and selective detection of Hg(ii) ions in aqueous solution with a low detection limit of 1.9 nM. Furthermore, the prepared colorimetric sensor was effectively applied for the quantification analysis of real water samples.

## Abstract 2

A novel colorimetric sensing platform using the peroxidase mimicking activity of ternary MoS_2_-loaded ZnO–g-C_3_N_4_ nanocomposites (ZnO–g-C_3_N_4_/MoS_2_) has been developed for the determination of Hg(ii) ions over co-existing metal ions.

## Graphical abstract

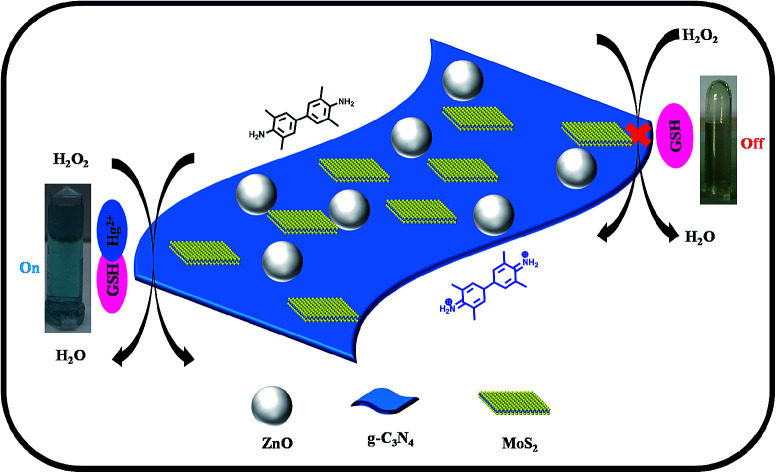

## 1. Introduction

Extensive industrial growth and modern agricultural practices in recent centuries have led to the contamination of natural ecosystems with various heavy metal ions. Among these, mercury is one of the most toxic heavy metal ions and is present in both elemental and organometallic complex forms in the natural aquatic environment.^1–4^ This water soluble, non-biodegradable divalent mercuric ion causes a wide range of adverse effects to the major organs and systems of the human body such as the brain, kidney, liver, skeletal, immune, nervous and cardiovascular systems.^5–9^ According to the World Health Organization (WHO), the guideline value of inorganic mercury in drinking water is 30 nM and the permissible level of Hg(ii) in natural water bodies was fixed as 10 nM by the United States Environmental Protection Agency (EPA).^10^ Since serious health problems arise from mercury contaminated water, the research community has shown a significant interest in developing a novel and sensitive analytical tool to detect this potential pollutant below the permissible level given by the EPA.

In the past few decades, the detection of mercury in natural water has gained considerable attention, and diverse conventional methods have been employed to detect trace levels of mercury in the aquatic environment such as atomic absorption spectroscopy (AAS), atomic fluorescence spectroscopy (AFS), inductively coupled plasma mass spectrometry (ICP-MS) and inductively coupled plasma atomic emission spectrometry (ICP-AES).^11–15^ However, all these methods have their own pros and cons; these methods need expensive instruments, complicated pretreatment methods, and highly skilful technicians and it is impossible to do on-site field detection and they are incapable of achieving the necessary detection limit due to the interference of other counter metal ions. To overcome these difficulties and make the detection process more simple and convenient, we need a novel method which is facile, low cost, easy to operate and highly sensitive with portable facilities for the *in situ* detection of Hg(ii) in aqueous solutions. To fulfil all these requirements, a colorimetric method is the one of the best when compared with all other detection methods. By means of the colorimetric sensor, we can rapidly detect the potential pollutant Hg(ii) with a marked colour change that can be visualized by the naked eye.^16–21^

Recently, much research has been done on the synthesis of novel nanocomposite materials that have enzyme mimicking catalytic activity, similar to that of natural enzymes such as catalyzes, oxidase, peroxidase, *etc.*^22^ These enzyme mimicking nanocomposite materials play a major role in the colorimetric sensing of heavy metals in aqueous solution, due to their intrinsic catalytic activity, high stability, ease of operation, and cost-effectiveness.^23,24^ Recently, it was shown that unanticipated peroxidase-like activity was exhibited by various inorganic nanoparticles (NPs) such as CeO_2_ NPs,^25^ Co_3_O_4_ NPs,^26^ Au NPs,^27^ MoS_2_ nanosheets^28^ and carbon-based nanomaterials like graphene oxide (GO),^29^ carbon nanotubes,^30^ carbon dots,^31^ carbon nitride^32^*etc.* Moreover, a number of hybrid nanocomposites were fabricated using metal nanomaterials and other matrixes such as GO–Fe_3_O_4_ (ref. 33), Pt–MoO_3_ (ref. 34) and Fe_3_O_4_@SiO_2_@Au@mSiO_2_ (ref. 35) and the intrinsic peroxidase mimicking activity was explored. It is an extremely challenging job to design a metal nanocomposite with excellent peroxidase like activity. ZnO shows exceptional catalytic properties and these can be further increased with suitable coupling to g-C_3_N_4_ nanosheets due to their large surface area and additional active sites on their edges. This hybridized nanocomposite further coupled with a suitable co-catalyst like MoS_2_ can accelerate the electron transfer at the interface of ZnO and g-C_3_N_4_. The hybridized nanocomposite produced by ZnO embedded in co-stacked g-C_3_N_4_/MoS_2_ nanosheets acts as a superior catalyst in peroxidase mimicking activity of the colorimetric detection.

In this study, we have designed a novel switch “off–on” colorimetric sensing system with ternary nanocomposite ZnO–g-C_3_N_4_/MoS_2_, tetramethyl benzidine (TMB), glutathione (GSH), hydrogen peroxide and sodium acetate buffer to detect Hg(ii) in an aqueous solution. The sensor turns “on” when the colour of the solution turns blue with the formation of TMB cation radicals due to the oxidation of TMB by ZnO–g-C_3_N_4_/MoS_2_ in the presence of hydrogen peroxide. When GSH is added into the solution, the blue colour disappears and the sensor turns “off”. This was due to the strong radical restoration ability of GSH. When Hg(ii) is introduced into the colorimetric system, GSH/ZnO–g-C_3_N_4_/MoS_2_/TMB, the sensor is switched “on” and the blue colour is regained in the solution due to the strong affinity of Hg(ii) with thiolated GSH. Based on this sensing strategy, we have newly employed a highly sensitive and selective colorimetric sensor for the detection of Hg(ii) in aqueous solutions. The above sensing strategy was successfully applied to real water samples, the promising results of the analysis proved that this can be applied to environmental applications.

## 2. Experimental section

### 2.1. Materials and methods

All the reagents and chemicals were purchased as analytical grade and can be used without any further purification. Zinc acetate dihydrate (Zn (Ac)_2_·2H_2_O), melamine, sodium molybdate, thiourea, Triton-X, sodium bicarbonate (NaHCO_3_), hydrogen peroxide (H_2_O_2_), sodium acetate, acetic acid, glutathione, and 3,3′,5,5-tetramethylbenzidine (TMB) were purchased from Alfa Aesar (India).

The following metallic salts CdCl_2_, FeCl_2_, FeCl_3_, MgCl_2_, ZnCl_2_, Pb(NO_3_)_2_, MnO_2_, NaCl, KCl, Cr(NO_3_)_3_, CaCO_3_, PO_4_^3−^, and SO_2_^2−^ were used to prepare stock solutions for the selectivity study and were bought from Sigma-Aldrich (India). Milli-Q water was used for the preparation of all the solutions in our studies. Organic solvents were purchased from Siscon Research Laboratories Pvt. Ltd (India).

The morphology and structure of the synthesized samples were analyzed using a field emission scanning electron microscope (FE-SEM), FEI Quanta FEG200, and transmission electron microscopy (TEM) analysis was measured using a JEOL/JEM 2100. For sample preparation, the powders were dispersed in ethanol, and a drop solution was dropped onto a 200 mesh carbon-coated TEM grid. X-Ray diffraction (XRD) was carried out using a PAN analytical X’pert powder Diffractometer using Cu-Kα radiation (*λ* = 0.15405 Å). Fourier transform infrared (FT-IR) spectra were recorded using a Nicolet 400 Fourier transform infrared spectrometer (Madison, WI), a Shimadzu, UV-2600 spectrophotometer was used for the measurement of UV visible spectra and a PHS-3C digital pH meter was used to check the pH values of the solutions.

### 2.2. Synthesis of g-C_3_N_4_ and MoS_2_

Bulk g-C_3_N_4_ was prepared based on a previously reported method.^36^ In this method, the melamine was heated at 550 °C in a muffle furnace for 4 h. The obtained yellow solid was allowed to cool at room temperature and then well ground in a mortar to get a fine powder of g-C_3_N_4_. Then it was washed with water, followed by ethanol and finally dried.

MoS_2_ was prepared according to an earlier reported procedure.^37^ In this method, 30 ml of aqueous sodium molybdate (6 mmol) and another 30 ml of thiourea (25 mmol) were prepared separately and both solutions were mixed together and sonicated for 30 min. The mixed solution was kept under magnetic stirring for 30 minutes and then transferred into a 100 ml Teflon-lined stainless steel autoclave and heated at 220 °C for 20 hours. After heating it was allowed to cool to room temperature. The obtained solid product was washed with distilled water followed by ethanol and dried at 60 °C for 10 h.

### 2.3. Exfoliation of MoS_2_ and g-C_3_N_4_

The MoS_2_ and g-C_3_N_4_ nanosheets were prepared by exfoliation of the bulk MoS_2_ and g-C_3_N_4_. MoS_2_ nanosheets were prepared by dispersing 50 mg of bulk MoS_2_ in 50 ml of mixed solvent (50 : 50 of ethanol : water) and sonicated for 3 h in an ice bath to control the temperature. Then the mixture was centrifuged to remove the heavier particles and the supernatant solution was dried to get the nanosheets. The same exfoliation method was followed for the preparation of g-C_3_N_4_ nanosheets.

### 2.4. Preparation of ZnO–g-C_3_N_4_/MoS_2_

Ternary nanocomposite ZnO–g-C_3_N_4_/MoS_2_ was prepared based on the wetness impregnation method. At first, 10 ml of Triton-X 100 (0.32 mmol L^−1^) was added into 100 ml of NaHCO_3_ (0.1 mol L^−1^) in a beaker and allowed to stir in a magnetic stirrer. In this solution, 100 ml of zinc acetate solution (0.1 mol L^−1^) was added in drops. After 60 minutes 0.6 mg of g-C_3_N_4_ nanosheets and 0.6 mg of MoS_2_ nanosheets were added successively. The mixture was continuously stirred for 12 h, and then heated to evaporate the solvent at 70 °C. The obtained powder was dried at 60 °C for 3 h in a hot air oven and then calcined at 200 °C for 3 h.

### 2.5. Determination of mercury ions in aqueous solution

Mercury ions in aqueous solutions were detected using the colorimetric method at room temperature. In this method, different concentrations of Hg(ii) were added separately into solutions containing GSH (100 μL) and sodium acetate buffer (pH = 3.5). The mixture was incubated at room temperature for 15 min. In this solution, 1 ml of 0.1 mM TMB and 500 μL of 0.008 mg ml^−1^ of ZnO–g-C_3_N_4_/MoS_2_ was added and the final volume of the solutions were made up to 2 ml and incubated for another 10 minutes. Finally, the absorption spectra were measured at 652 nm for all samples with a UV-Vis spectrometer.

## 3. Result and discussion

### 3.1. Characterization

**Fig. 1:**
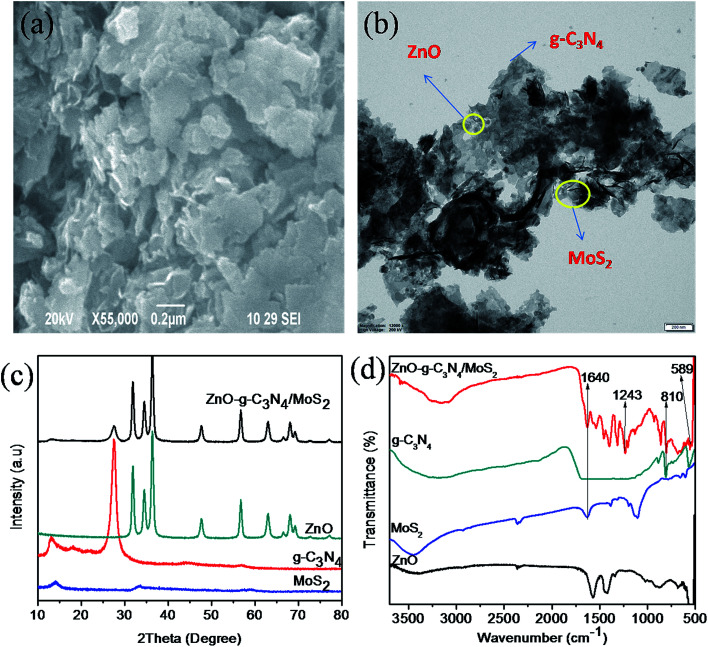
SEM image (a) and TEM image (b) of ZnO–g-C_3_N_4_/MoS_2_, XRD patterns (c) and FT-IR spectra (d) of MoS_2_, g-C_3_N_4_, ZnO and ZnO–g-C_3_N_4_/MoS_2_.

The surface morphology of the synthesized ternary nanocomposite investigated using Field Emission Scanning Electron Microscopy (FESEM), as shown in (Fig. 1a), inferred that thin layers of g-C_3_N_4_ and MoS_2_ nanosheets were stacked over ZnO. Transmission electron microscopy (TEM) analysis, shown in (Fig. 1b), confirmed that the stacked g-C_3_N_4_ and MoS_2_ nanosheets provide more interfacial contact with ZnO between g-C_3_N_4_ and MoS_2_ nanosheets. The presence of elements such as Zn, O, C, N, Mo, and S present in the ternary nanocomposite was confirmed using EDS and EDS mapping analysis (Fig. S1 and S2†). The XRD patterns of pure materials like MoS_2_, g-C_3_N_4_, ZnO and the composite ZnO–g-C_3_N_4_/MoS_2_ are shown in Fig. 1c. A single tiny diffraction peak at 2*θ* = 13.9 represents the MoS_2_, the diffraction peak observed at 2*θ* = 27.2° is the characteristic peak of pure g-C_3_N_4_. The diffraction peaks of pure ZnO with 2*θ* = 31.2°, 34.8°, 36.8°, 47.6°, 56.7°, 63.4°, 67.8° and 68.1° were matched with the standard data (JPCDS-36-1451). The diffraction pattern of the ZnO–g-C_3_N_4_/MoS_2_ hybrid composite was similar to that of ZnO. However, there were small diffraction peaks observed at 2*θ* = 13.8° and 27.2° assigned to the characteristic peaks of MoS_2_, g-C_3_N_4_ respectively. From the XRD patterns, it was clear that stacking a few layers of MoS_2_ and g-C_3_N_4_ on ZnO will not affect the structure of ZnO. Fourier transform infrared (FTIR) spectra (Fig. 1d) were recorded for ZnO, MoS_2_, g-C_3_N_4_, and the ZnO–g-C_3_N_4_/MoS_2_ ternary nanocomposite in the range of 500–3750 cm^−1^. The peak observed at 558 cm^−1^ corresponds to the bending vibrations of Zn–O. The peaks observed at 810 cm^−1^ for g-C_3_N_4_ and ZnO–g-C_3_N_4_/MoS_2_ correspond to the *s*-triazine ring of g-C_3_N_4_ and the strong interaction between ZnO and g-C_3_N_4_ in the case of ZnO–g-C_3_N_4_/MoS_2_. The peak observed at 1243 cm^−1^ for ZnO–g-C_3_N_4_/MoS_2_ is assigned to aromatic C–N stretching. The broad band recorded in the range of 3000–3500 cm^−1^ is assigned to the hydroxyl group of H_2_O. From the FTIR spectral analysis it was confirmed that the obtained ternary nanocomposite was ZnO–g-C_3_N_4_/MoS_2_.

### 3.2. Peroxidase-like activity of ZnO–g-C_3_N_4_/MoS_2_ nanocomposites

**Fig. 2:**
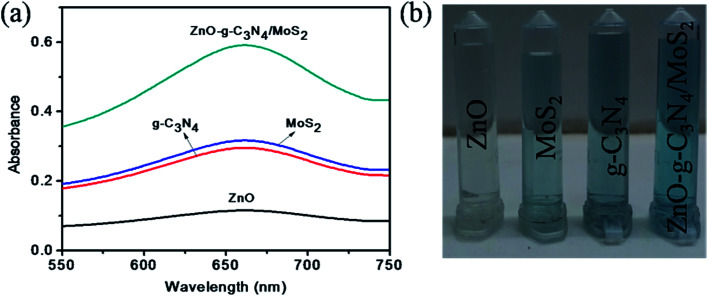
(a) UV-Visible absorption spectra of ZnO, MoS_2_, g-C_3_N_4_, and ZnO–g-C_3_N_4_/MoS_2_ in the presence of TMB, sodium acetate buffer and H_2_O_2_ and (b) photographic picture of the test samples.

A study was made to determine the peroxidase mimicking catalytic activity of ZnO–g-C_3_N_4_/MoS_2_ nanocomposites using TMB as a peroxidase substrate. The UV-Vis absorption spectrum shown in Fig. 2a indicates that with TMB in the presence of H_2_O_2_, the ZnO did not show any characteristic absorption, and for g-C_3_N_4_ and MoS_2_ it shows a moderate absorption at 652 nm. However, for ZnO–g-C_3_N_4_/MoS_2_ a strong absorption peak was observed at 652 nm. These results confirm that the ZnO–g-C_3_N_4_/MoS_2_ nanocomposite possesses good catalytic activity for the oxidation of TMB molecules in the presence of H_2_O_2_. Fig. 2b shows a photographic image of the sample solutions used for the measurement of the absorption spectra.

### 3.3. Sensing mechanism of the Hg(ii)

**Fig. 3:**
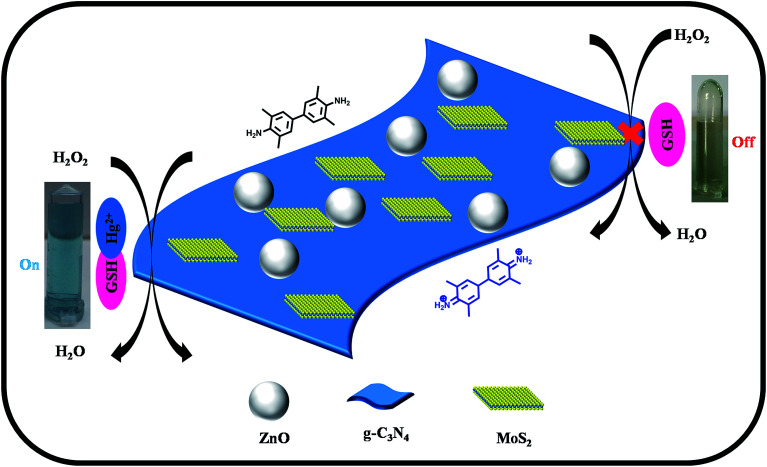
Schematic diagram for the colorimetric detection of Hg(ii) ions based on the ZnO–g-C_3_N_4_/MoS_2_ ternary nanocomposite.

The scheme in Fig. 3 represents the peroxidase mimicking mechanism of the ZnO–g-C_3_N_4_/MoS_2_-based colorimetric assay for the detection of Hg(ii) ions in aqueous solution. The ZnO–g-C_3_N_4_/MoS_2_ composite was designed by embedding ZnO nanospheres on the co-stacked g-C_3_N_4_/MoS_2_ nanosheets. The ZnO nanospheres coupled with g-C_3_N_4_/MoS_2_ nanosheets possess excellent catalytic activity for the oxidation of TMB into its radical cation (TMB^+^) to produce a deep blue colour in presence of H_2_O_2_ solution. The addition of GSH faded the solution colour due to its strong cation restoration properties. When Hg(ii) was introduced into the same solution, a strong complex formed between Hg(ii) and the thiol group of GSH, which brought the deep blue colour back into the solution. The interaction between GSH and Hg(ii) in our mechanism was confirmed with the FT-IR spectra shown Fig. S3.† While comparing the GSH spectrum with that of GSH–Hg, the thiol peak which appeared at 2480 cm^−1^ in the GSH spectrum disappeared in GSH–Hg spectrum, with the addition of a sharp narrow peak appearing at 1629 cm^−1^ due to Hg–S interactions.

**Fig. 4:**
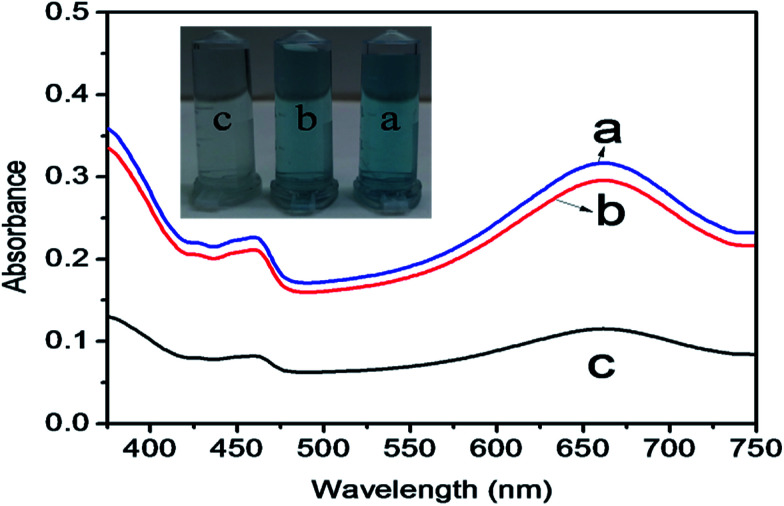
Absorption spectra (a) TMB + ZnO–g-C_3_N_4_/MoS_2_ + GSH + Hg^2+^ (blue line), (b) TMB + ZnO–g-C_3_N_4_/MoS_2_ (red line), and (c) TMB + ZnO–g-C_3_N_4_/MoS_2_ + GSH (black line). Inserted picture represents the colouration of the three samples.

**Fig. 5:**
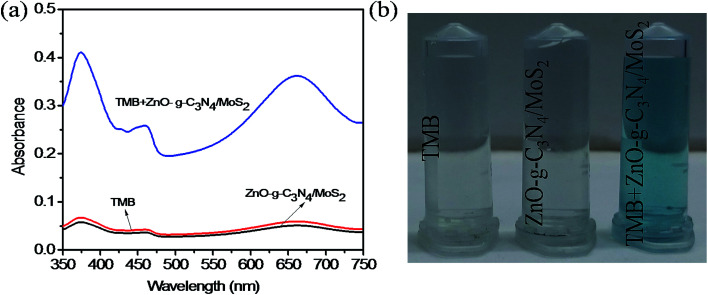
(a) The absorption spectra of the TMB (red line), ZnO–g-C_3_N_4_/MoS_2_ (black line), and mixed TMB + ZnO–g-C_3_N_4_/MoS_2_, (blue line), (b) the photographic picture corresponds to the analytical samples.

The mechanism of colorimetric sensing of Hg(ii) in aqueous solution was investigated with three systems (a, b, c), (a) TMB + ZnO–g-C_3_N_4_/MoS_2_ + GSH + Hg^2+^, (b) TMB + ZnO–g-C_3_N_4_/MoS_2_ and (c) TMB + ZnO–g-C_3_N_4_/MoS_2_ + GSH. The change in the colour of the systems was visually detected and observed in the UV-Vis absorption spectra at 652 nm. Fig. 4 shows that the absorption peak observed at 652 nm for (a) and (b) and the change in colour of the solution into blue was due to the strong affinity of GSH with Hg(ii) in system (a) and the catalytic oxidation of TMB by the substrate nano composite ZnO–g-C_3_N_4_/MoS_2_ in system (b). System (c) does not show any characteristic peak at 652 nm, and the solution appears colourless due to the strong cation restoration ability of GSH by its thiol functionality. The inserted photograph picture in Fig. 4 represents the analytical samples (a, b, c). Further absorption spectra were taken for solutions containing TMB and ZnO–g-C_3_N_4_/MoS_2_ and a mixed solution of TMB + ZnO–g-C_3_N_4_/MoS_2_, as shown in Fig. 5. It was observed that an absorption peak occurs at 652 nM only in the case of the solution containing both TMB and ZnO–g-C_3_N_4_/MoS_2_. No absorption peaks were obtained when the solution contains TMB and ZnO–g-C_3_N_4_/MoS_2_ alone. From the absorption spectra, it was clear that oxidation takes place only when the solution contains both TMB and ZnO–g-C_3_N_4_/MoS_2_. The ZnO–g-C_3_N_4_/MoS_2_ nanocomposite in the presence H_2_O_2_ in the solution catalyzed the TMB into its radical ions (TMB^+^). The photographic picture (Fig. 5b) shows the analytical samples taken for the absorption spectra.

### 3.4. Optimization of experimental conditions

**Fig. 6:**
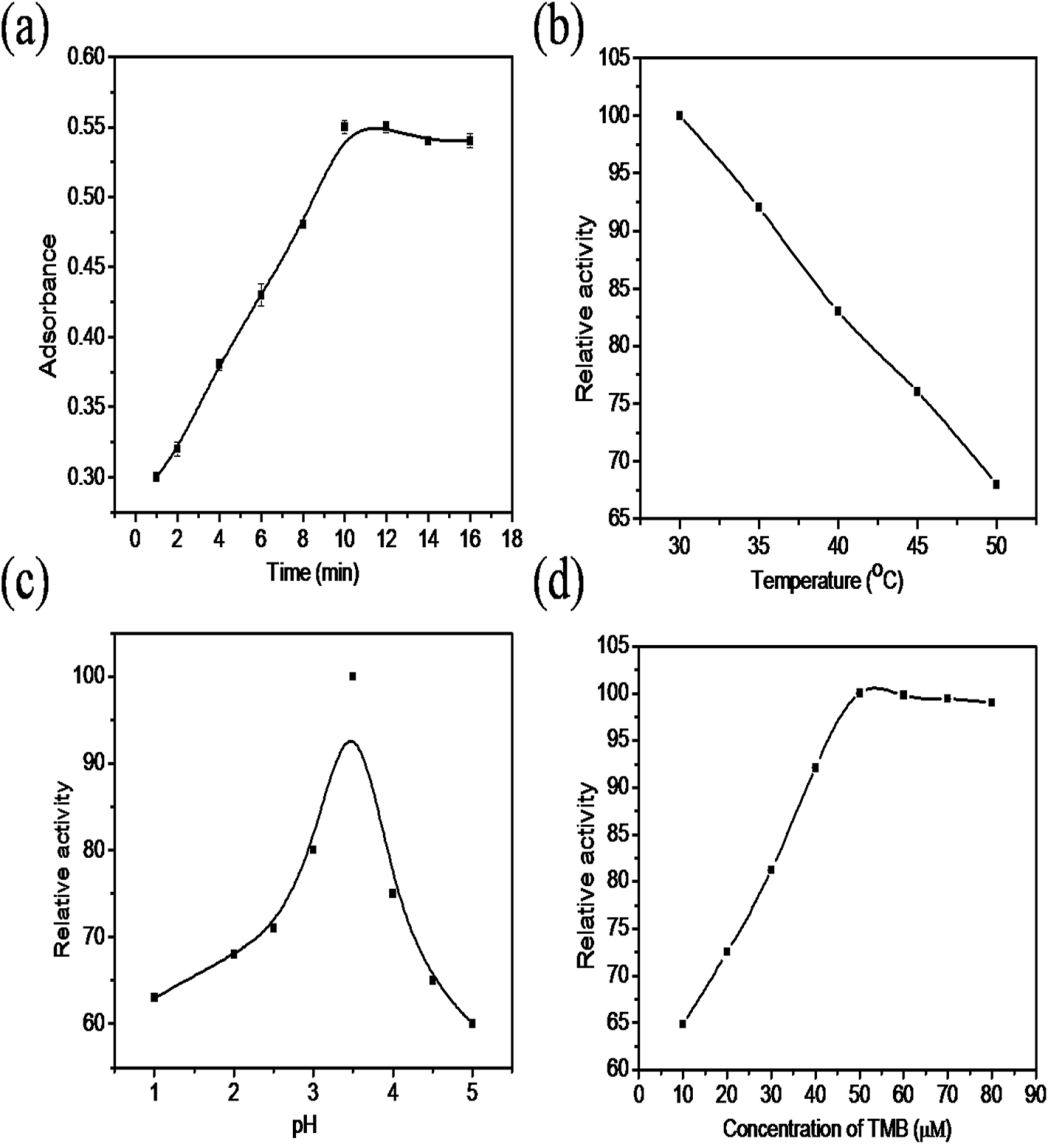
The effect of the (a) incubation time (b) temperature, (c) pH, and (d) concentration of the TMB.

The colorimetric sensing performance of the synthesized nanocomposite was studied under different reaction conditions such as changes in time, temperature, solution pH and concentration of TMB. Fig. 6a shows that the absorbance increases rapidly as the reaction time increases from 0 to 10 minutes. After 10 minutes there was no change in absorbance noticed because, in the initial stage, the availability of TMB is more in the solution, so the absorbance drastically increases due to oxidation, after 10 minutes most of the TMB molecules were oxidized into TMB^+^. The effect of reaction temperature is indicated in Fig. 6b, the absorbance value decreases as temperature increases from 30 °C to 50 °C due to the instability of H_2_O_2_ at higher temperature. The relative activity of the nanocomposite at different pH values is shown in Fig. 6c. The relative activity increased as the pH of the solution increases from 1 to 3.5 and then decreases due to the instability of H_2_O_2_ at higher pH. The relative activity of the ternary nanocomposite increases as the concentration of TMB increases from 10–50 μM (Fig. 6d) and then remains constant. This is may be due to unavailability of oxidant for the oxidation of the TMB in the solution. Based on these results, it was decided to optimize the reaction parameters such as time, temperature, pH and TMB concentration as 10 min, 30 °C, 3.5 and 50 μM, respectively.

### 3.5. Colorimetric detection of Hg(ii)

**Fig. 7:**
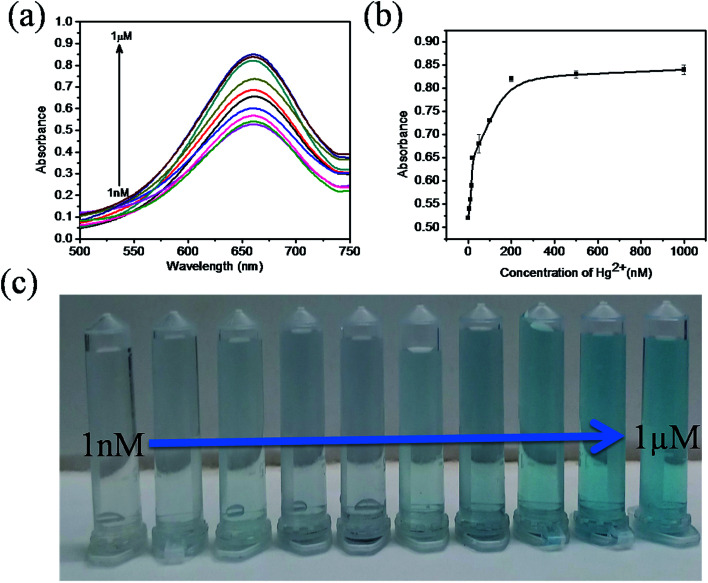
Absorption spectra of Hg(ii) in aqueous solutions, (a) the UV-Vis spectra of sensing system (TMB + ZnO–g-C_3_N_4_/MoS_2_ + GSH + H_2_O_2_) with different concentrations of Hg(ii) ions, (b) graph plot of absorbance values with respect to the increasing concentrations of Hg(ii), (c) photographic image of Hg(ii) ion sample solutions with the concentration range 1 nM to 1 μM.

**Fig. 8:**
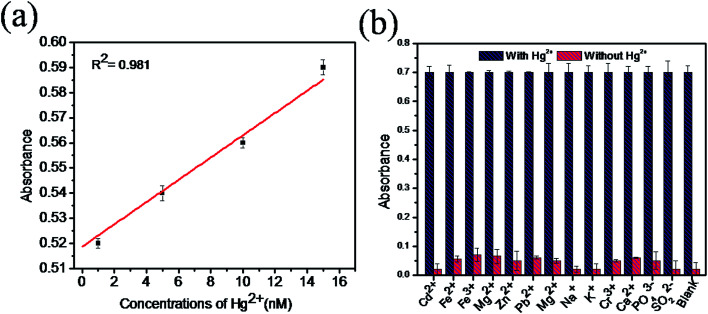
(a) The linear calibration plot of absorbance in the concentration range of 1–15 nM of mercury, (b) absorption plot of various metal ions with and without the presence of Hg(ii) ions for selectivity determination of ZnO–g-C_3_N_4_/MoS_2_.

The sensitivity of Hg(ii) in aqueous solutions was investigated using the colorimetric method under optimized conditions with different concentrations of Hg(ii) in the range of 0 to 1 μM. Fig. 7a clearly reveals that the absorbance value of the sensing system in the UV spectra at 652 nm gradually increases as the concentration of Hg(ii) increases from 0 to 1 μM. As the concentration of Hg(ii) in the solution increases, the affinity between Hg(ii) and GSH increases. This retards the cation restoration properties of the GSH and increases the catalytic oxidation of TMB into TMB radical ions and intensifies the blue colour in the solution. Fig. 7b indicates the relationship between the absorbance intensity and concentration of Hg(ii) and Fig. 7c shows the photographic picture of Hg(ii) ion solutions with the concentration range 1 nM to 1 μM. A good linear correlation coefficient (*R*^2^ = 0.981) exists between the absorbance intensity and concentration of Hg(ii) in the range of 1–15 nM, as shown in Fig. 8a, and the limit of detection of Hg(ii), calculated using the 3σ method, was 1.9 nM. Comparisons of the detection limit values of various nanomaterial based colorimetric sensors with our sensor are listed in Table 1.

**Table 1:** Comparison of various nanomaterial-based colorimetric methods of Hg^2+^ detection

Materials	Detection limit	Real samples	Reference
ZnO–g-C_3_N_4_/MoS_2_	1.9 nM	Tap/well/pond water	Present work
MnO_2_	0.8 μM	Pool/river water	23
Graphene–gold nanocomposite	16 nM	Tap/well/river water	38
CoS-p-rGO	14.23 nM	Tap/well/pond/river water	39
Silver nanoparticles	2.73 nM	Tap/lake/drinking water	40
Gold NPs	8 nM	Pond water	41
Carbon nanodots	23 nM	River water	42

### 3.6. Selectivity for Hg^2+^ of the proposed colorimetric sensing system

The selectivity for Hg(ii) of our sensing system was studied with samples containing different metal ions such as Cd^2+^, Fe^2+^, Fe^3+^, Mg^2+^, Zn^2+^, Pb^2+^, Mn^2+^, Na^+^, K^+^, Cr^3+^, PO_4_^3−^, SO_3_^2−^ and Pb^2+^ with Hg(ii) (5 μM) and without Hg(ii) under optimal conditions. Fig. 8b shows that all the metal ion samples with mercury show good and equal amounts of absorbance, whereas without mercury, very low absorbance was observed. This result confirms that the presence of coexisting metal ions in the solution does not interfere with the absorption of mercury. Therefore, the proposed colorimetric sensing system is highly selective in the determination of mercury in aqueous solutions.

### 3.7. Detection of Hg(ii) ions in real samples

Detection of Hg^2+^ in real water samples was studied using our colorimetric sensing system under optimal conditions with water samples collected from different sources around the SRM Institute of Science and Technology campus such as tap, well, and lake water. The suspended impurities in the water samples were first filtered with a 0.22 micrometer membrane and the pH was adjusted to 3.5 with sodium acetate buffer. Since the environmental samples do not contains Hg^2+^, different concentrations of Hg(ii) were spiked into the water samples and the recovery of mercury ions was measured using standard methods. The analytical results are displayed in Table 2. All the types of sample were measured thrice, and the results confirmed that our proposed sensing method is highly sensitive and selective in the determination of Hg(ii) in real water analysis for environmental monitoring.

**Table 2:** Results of recovery values from the analysis of Hg^2+^ in real water samples

Samples	Hg^2+^ added (nM)	Hg^2+^ found (nM)	Recovery (%)	RSD (%)
Tap water	10	9.94	99.4	0.46
	25	24.6	98.4	0.38
	50	49.6	99.2	0.64
Well water	10	9.88	98.8	0.40
	25	24.2	96.8	0.68
	50	49.7	99.4	0.52
Pond water	10	9.93	99.3	0.38
	25	24.4	97.6	0.49
	50	48.7	97.4	0.70

## 4. Conclusion

In precis, a novel colorimetric sensing system was developed to detect Hg(ii) in aqueous solution using the hybrid ZnO–g-C_3_N_4_/MoS_2_ nanocomposite as a peroxidase mimicking sensor with TMB and H_2_O_2_. The colorimetric signals were produced by the generation of a blue colour in the test solution due to the oxidation of TMB, and vanishing of the colour was due to the restoration of TMB^+^ by GSH and the regeneration of the blue colour was due to complex formation between Hg(ii) and GSH. The sensing mechanism was favored in acidic media with a detection limit value of 1.9 nM, with a linear relationship of *R*^2^ = 0.981. This colorimetric assay was successfully applied to detect Hg(ii) in real water samples. We expect that this strategy may offer a new approach in developing a selective sensor for monitoring Hg(ii) in environmental samples.

## Conflicts of interest

There are no conflicts to declare.

## Supplementary Material

RA-009-C8RA09814J-s001
