# Supplementary material for: MoS2 nanosheet mediated ZnO–g-C3N4 nanocomposite as a peroxidase mimic: catalytic activity and application in the colorimetric determination of Hg(ii)
Source: RSC Adv. 2019 Feb 1;9(8):4268–76. doi: 10.1039/c8ra09814j (PMC9060464; doi:10.1039/c8ra09814j)
Supplement: RA-009-C8RA09814J-s001 [file RA-009-C8RA09814J-s001.pdf]

Electronic Supplementary Material (ESI) for RSC Advances.
This journal is © The Royal Society of Chemistry 2019

Supplementary Materials

MoS_2 Nanosheets Mediated ZnO–g-C_3N_4 Nanocomposite as a Peroxidase Mimics Catalytic Activity and its Application to the Colorimetric Determination of Hg (II) Sensor

A. Anand Babu Christus[1], P. Panneerselvam[2*], A.Ravikumar[2], M. Marieeswaran[2], S. Sivanesan[3]

[1]Department of Chemistry, SRM Institute of Science and Technology, Ramapuram campus,

Ramapuram- 600 089, Tamil Nadu, India.

[2]Department of Chemistry, SRM Institute of Science and Technology, Kattankulathur- 603 203,

Tamil Nadu, India.

[3]Department of Applied Science and Technology, A. C. Tech Campus, Anna University,

Chennai-600 025

Correspondingaddressand Email address: panneerselvam.pe@ktr.srmuniv.ac.in,

panneerchem82@gmail.com

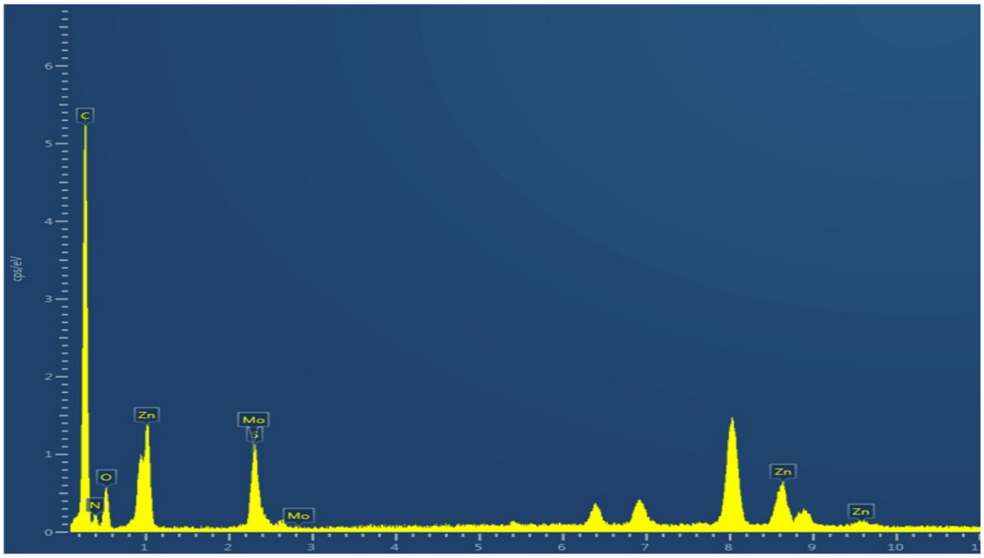

Fig S1 EDX spectrum of ZnO–g-C_3N_4/MoS_2 nanocomposite

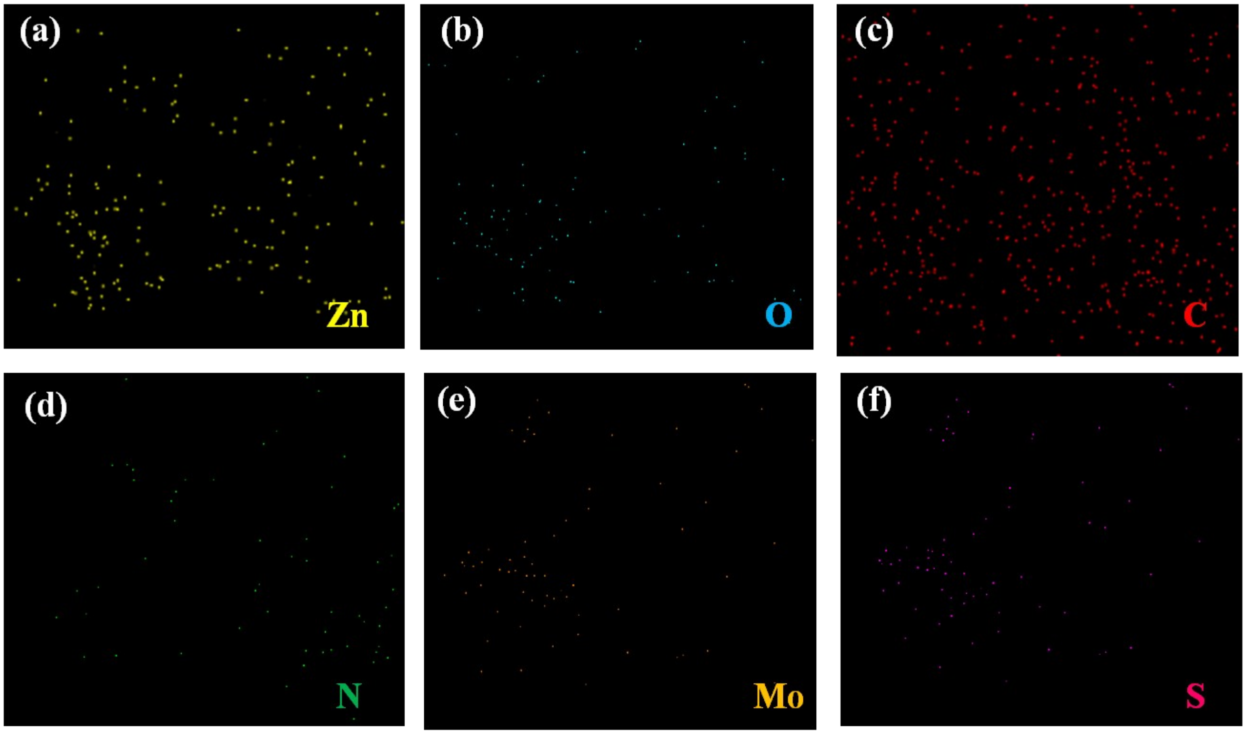

Fig. S2: EDS mapping analysis of the ZnO–g-C_3N_4/MoS_2 nanocomposite containing Zn(a), O(b), C (c), N (d), Mo (e), and S (f).

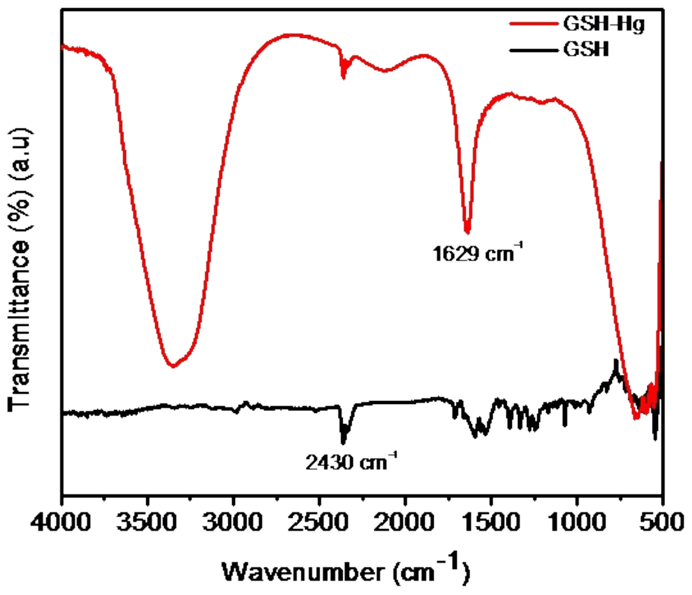

Fig. S3 FT-IR spectra of pure GSH and GSH-Hg
